# Detecting multivariate differentially expressed genes

**DOI:** 10.1186/1471-2105-8-150

**Published:** 2007-05-09

**Authors:** Roland Nilsson, José M Peña, Johan Björkegren, Jesper Tegnér

**Affiliations:** 1 Computational Biology, Department of Physics, Linköping University, SE-581 53 Linköping, Sweden; 2 Unit of Computational Medicine, King Gustaf V Research Institute, Department of Medicine, Karolinska Institutet, SE-171 76 Stockholm, Sweden

## Abstract

**Background:**

Gene expression is governed by complex networks, and differences in expression patterns between distinct biological conditions may therefore be complex and multivariate in nature. Yet, current statistical methods for detecting differential expression merely consider the univariate difference in expression level of each gene in isolation, thus potentially neglecting many genes of biological importance.

**Results:**

We have developed a novel algorithm for detecting multivariate expression patterns, named Recursive Independence Test (RIT). This algorithm generalizes differential expression testing to more complex expression patterns, while still including genes found by the univariate approach. We prove that RIT is consistent and controls error rates for small sample sizes. Simulation studies confirm that RIT offers more power than univariate differential expression analysis when multivariate effects are present. We apply RIT to gene expression data sets from diabetes and cancer studies, revealing several putative disease genes that were not detected by univariate differential expression analysis.

**Conclusion:**

The proposed RIT algorithm increases the power of gene expression analysis by considering multivariate effects while retaining error rate control, and may be useful when conventional differential expression tests yield few findings.

## Background

**Figure 1:**
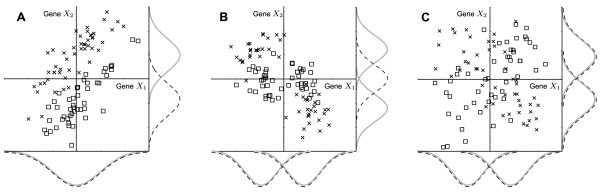
**Distribution example**. Example two-gene distributions with multivariate effects. Each square/cross denotes a sample from one of the classes. Black dashed/gray solid lines denote the corresponding class-conditional marginal distributions. **A**: A PCWT distribution where the gene *X*_2 _is UDE (since its class-conditional means differ) while *X*_1 _is not. **B**: A PCWT distribution where neither gene is UDE (and hence cannot be detected by standard differential expression tests). **C**: A non-PCWT distribution. In all cases, both genes are MDE.

The problem of detecting a change in expression between two biological states, such as healthy *vs. *diseased, is central to microarray data analysis. There are two main approaches to this problem: statistical difference tests [1] or feature selection by machine learning techniques [2]. The former alternative provides a solid statistical foundation and allows proper control of false positive rates, but is limited to detecting differences in the expression level of single genes. We herein refer to this as *univariate differential expression *(UDE). The machine learning techniques on the other hand can potentially discover more complex, multivariate effects, herein referred to as *multivariate differential expression *(MDE). Figure 1 provides some examples illustrating the distinction between UDE and MDE. However, machine learning techniques usually aim to discover small, predictive gene sets and do not control error rates. Consequently, the gene lists obtained are often unreliable [3,4]. Thus, there is a need for novel methods that combine the best of the two worlds – allowing detection of MDE patterns within a sound statistical framework.

Existing solutions to this problem center around the idea of performing statistical tests on *gene sets *rather than on individual genes. Examples of this include the popular Gene Set Enrichment Analysis [5] method and various multivariate tests [6,7]. This approach permits detection of multivariate effects, but it requires the user to choose the gene sets involved in advance. The latter simplifies the problem considerably, but consequently only permits detection of previously known functional groups such as KEGG pathways [8] or GeneOntology categories [9].

Earlier, Szabo et al. [10] proposed to find novel gene sets by maximizing a test statistic *D *using subset search. Unfortunately, since exhaustive subset search is intractable for high-dimensional data, Szabo et al. are forced to resort to heuristics, considering only gene sets of some *a priori *fixed size and using randomized search methods. Xiao et al. [11] developed this procedure further and used a permutation test to ensure that *D *is significantly larger than what would be expected by random. In this way the error rate *over gene sets *can be controlled, at least approximately. Dettling et al. [12] proposed a different approach for finding gene sets, but only considered sets of size 2 and restricted attention to certain types of bivariate normal distributions.

In our view, a limitation with all of the above methods is that the error rates necessarily concern gene sets, not individual genes. Since a gene set may be significant even when containing only a single true positive gene [10], the false positive rate over individual genes may be much higher than the false positive rate over gene sets. For example, if a single, true gene set containing 10 genes is selected, then the false positive rate over gene sets is zero, while the false positive rate over the genes involved may be as high as 90%. This is appropriate if the investigator is indeed primarily interested in gene sets; but if the primary interest is individual genes, then these methods may be misleading.

In this paper we focus on finding individual MDE genes, not gene sets. We present a principled, generally applicable method which can be seen as a direct generalization of univariate differential expression to the multivariate case. We prove that our procedure is *consistent *(i.e., given enough samples, it discovers exactly the true MDE genes) for a realistic class of data distributions. Further, we show that our method produces correct p-values for small samples, and thus controls error rates while offering more power than univariate differential expression testing. Finally, we apply our method to two microarray data sets and conduct a literature validation of the gene lists generated, revealing many potentially important genes that were not detected by the UDE tests.

### Theoretical background

Throughout, we assume that samples (arrays) *x*^(1)^,... *x*^(*l*) ^are independent observations of an *n*-dimensional random vector *X *= (*X*_1_,..., *X*_*n*_), with class-conditional density *f*(*x*|*y*), where *y *∈ {-1, +1} is a class variable denoting the biological condition. For simplicity we here restrict ourselves to the two-class case, although the theory and methods presented can easily be extended to multiple classes or even continuous *Y*. For microarray data, we typically have *n *≫ *l*.

We say that a gene *X*_*i *_is *univariate differentially expressed *(UDE) if the mean expression level for the two conditions differ. Thus we define the set of UDE genes

*U *= {*X*_*i *_: E
 MathType@MTEF@5@5@+=feaafiart1ev1aaatCvAUfKttLearuWrP9MDH5MBPbIqV92AaeXatLxBI9gBaebbnrfifHhDYfgasaacH8akY=wiFfYdH8Gipec8Eeeu0xXdbba9frFj0=OqFfea0dXdd9vqai=hGuQ8kuc9pgc9s8qqaq=dirpe0xb9q8qiLsFr0=vr0=vr0dc8meaabaqaciaacaGaaeqabaqabeGadaaakeaatuuDJXwAK1uy0HMmaeHbfv3ySLgzG0uy0HgiuD3BaGabaiab=ri8fbaa@388C@[*X*_*i *_| *Y *= +1] ≠ E
 MathType@MTEF@5@5@+=feaafiart1ev1aaatCvAUfKttLearuWrP9MDH5MBPbIqV92AaeXatLxBI9gBaebbnrfifHhDYfgasaacH8akY=wiFfYdH8Gipec8Eeeu0xXdbba9frFj0=OqFfea0dXdd9vqai=hGuQ8kuc9pgc9s8qqaq=dirpe0xb9q8qiLsFr0=vr0=vr0dc8meaabaqaciaacaGaaeqabaqabeGadaaakeaatuuDJXwAK1uy0HMmaeHbfv3ySLgzG0uy0HgiuD3BaGabaiab=ri8fbaa@388C@[*X*_*i *_| *Y *= -1]}.

In figure 1A, this holds for gene *X*_2 _but not for gene *X*_1_; in figures 1B,C neither gene is UDE. In higher dimensions, similar situations may render a large fraction of the genes MDE yet not UDE. A more general definition is the following: we say that a gene *X*_*i *_is *multivariate differentially expressed *(MDE) if there exists a gene set *S *⊂ {*X*_1_,... *X*_*n*_} such that *X*_*i *_is conditionally dependent on *Y *given *S*. Thus the set of MDE genes is defined formally as

*M *= {*X*_*i *_: ∃*S *⊆ *X *: *X*_*i *_⊥
 MathType@MTEF@5@5@+=feaafiart1ev1aaatCvAUfKttLearuWrP9MDH5MBPbIqV92AaeXatLxBI9gBaebbnrfifHhDYfgasaacH8akY=wiFfYdH8Gipec8Eeeu0xXdbba9frFj0=OqFfea0dXdd9vqai=hGuQ8kuc9pgc9s8qqaq=dirpe0xb9q8qiLsFr0=vr0=vr0dc8meaabaqaciaacaGaaeqabaqabeGadaaakeaacuGHLkIxgaGcaaaa@2E6E@*Y *| *S*}.

where ⊥
 MathType@MTEF@5@5@+=feaafiart1ev1aaatCvAUfKttLearuWrP9MDH5MBPbIqV92AaeXatLxBI9gBaebbnrfifHhDYfgasaacH8akY=wiFfYdH8Gipec8Eeeu0xXdbba9frFj0=OqFfea0dXdd9vqai=hGuQ8kuc9pgc9s8qqaq=dirpe0xb9q8qiLsFr0=vr0=vr0dc8meaabaqaciaacaGaaeqabaqabeGadaaakeaacuGHLkIxgaGcaaaa@2E6E@ denotes conditional dependence. This definition recognizes all genes in figures 1A,B,C as MDE. It was initially proposed by Kohavi and John [13]. Clearly, the criterion (1) implies criterion (2), so we have *U *⊆ *M*. Therefore MDE is a generalization of UDE. Genes which are MDE but not UDE cannot be detected by univariate differential expression tests, as shown in figure 1. The goal of this paper is to estimate the set *M *from expression data.

The set *M *is typically larger than the set of genes optimal for *predicting Y*, because some genes in *M *may be "redundant" – their predictive information can be obtained from other genes, and hence they can safely be excluded from the predictor [13]. Therefore, machine learning techniques that attempt to optimize a predictor (such as the Recursive Feature Elimination [14] used herein) tend to select only a subset of *M*. Hence, these methods are generally not suitable for our purpose.

Unfortunately, for *arbitrary *data distributions, determining whether equation (2) holds for a given gene *X*_*i *_requires exhaustive subset search, which is known to be intractable for high-dimensional data. However, for a large class of data distributions we herein refer to as the *PCWT *class (short for Positive/Composition/Weak Transitivity; see Additional file 1 for a rigorous definition), we will prove that the problem is tractable. We will then show that this PCWT class is sufficiently general to be used as a model for biological data, and in particular microarray data.

## Results

### The RIT algorithm

**Figure 2:**
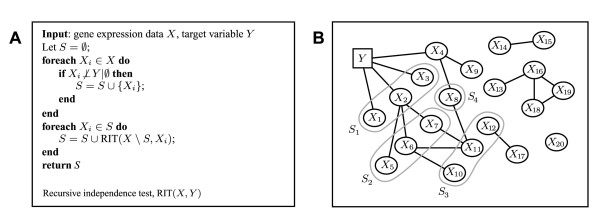
**The RIT Algorithm**. **A**: Algorithm pseudocode. **B**: Algorithm example. Edges (solid lines) denote marginal dependencies between genes *X*_*i *_(circles) and the class label variable *Y *(square). Gene sets found in each round of RIT are denoted *S*_1_,..., *S*_4_. The final output of the algorithm is the union of these.

We developed a recursive algorithm named Recursive Independence Test (RIT) based on pairwise tests for marginal independencies. The algorithm pseudocode is given in figure 2A. In the first round, RIT tests for the marginal independencies *X*_*i *_⊥ *Y *| ∅ for each gene *X*_*i *_and obtains a gene set *S *of significant findings. Next, for each *X*_*i *_∈ *S *we recursively call RIT to test for the marginal independencies *X*_*i *_⊥ *X*_*j *_| ∅ against each gene *X*_*j *_∉ *S*, and add the significant findings to *S*. We continue in this fashion until no more dependencies are found.

An illustrating example of an RIT run is given in figure 2B. Here, the MDE genes are *M *= {*X*_1_,..., *X*_11_}, the UDE genes are *U *= {*X*_1_,..., *X*_4_}, and the remaining genes are unrelated to *Y*. In the first round of RIT we obtain the set *S*_1_. In this case *S*_1 _differs from *U*, which of course may happen for small sample sizes since the statistical tests used have limited power. In the next recursion, RIT tests the genes in *S*_1 _against *X *\ *S*_1 _= {*X*_4_,..., *X*_20_}; this discovers the set *S*_2_, which is dependent on *X*_2_. Continuing the recursion, RIT eventually finds two more gene sets *S*_3_, *S*_4_, after which no more significant genes are found and the algorithm terminates. The final output of RIT is then the estimate M^
 MathType@MTEF@5@5@+=feaafiart1ev1aaatCvAUfKttLearuWrP9MDH5MBPbIqV92AaeXatLxBI9gBaebbnrfifHhDYfgasaacH8akY=wiFfYdH8Gipec8Eeeu0xXdbba9frFj0=OqFfea0dXdd9vqai=hGuQ8kuc9pgc9s8qqaq=dirpe0xb9q8qiLsFr0=vr0=vr0dc8meaabaqaciaacaGaaeqabaqabeGadaaakeaacuWGnbqtgaqcaaaa@2DDF@ = *S*_1 _∪ *S*_2 _∪ *S*_3 _∪ *S*_4_. In *S*_3 _we obtain a false positive *X*_12_, and since *X*_4 _∉ *S*_1_, we also fail to detect *X*_9 _because the required test is never made. Since the RIT algorithm only visits each *X*_*i *_∈ M^
 MathType@MTEF@5@5@+=feaafiart1ev1aaatCvAUfKttLearuWrP9MDH5MBPbIqV92AaeXatLxBI9gBaebbnrfifHhDYfgasaacH8akY=wiFfYdH8Gipec8Eeeu0xXdbba9frFj0=OqFfea0dXdd9vqai=hGuQ8kuc9pgc9s8qqaq=dirpe0xb9q8qiLsFr0=vr0=vr0dc8meaabaqaciaacaGaaeqabaqabeGadaaakeaacuWGnbqtgaqcaaaa@2DDF@ once, it is easy to see that the number of tests made is on the order of |M^
 MathType@MTEF@5@5@+=feaafiart1ev1aaatCvAUfKttLearuWrP9MDH5MBPbIqV92AaeXatLxBI9gBaebbnrfifHhDYfgasaacH8akY=wiFfYdH8Gipec8Eeeu0xXdbba9frFj0=OqFfea0dXdd9vqai=hGuQ8kuc9pgc9s8qqaq=dirpe0xb9q8qiLsFr0=vr0=vr0dc8meaabaqaciaacaGaaeqabaqabeGadaaakeaacuWGnbqtgaqcaaaa@2DDF@|*n*. Thus, for reasonably small M^
 MathType@MTEF@5@5@+=feaafiart1ev1aaatCvAUfKttLearuWrP9MDH5MBPbIqV92AaeXatLxBI9gBaebbnrfifHhDYfgasaacH8akY=wiFfYdH8Gipec8Eeeu0xXdbba9frFj0=OqFfea0dXdd9vqai=hGuQ8kuc9pgc9s8qqaq=dirpe0xb9q8qiLsFr0=vr0=vr0dc8meaabaqaciaacaGaaeqabaqabeGadaaakeaacuWGnbqtgaqcaaaa@2DDF@, the algorithm scales approximately linearly with the number of genes. This is important not only for computational speed, but also to reduce multiplicity problems (see below). Note also that since the first round of RIT is a univariate differential expression test, the set M^
 MathType@MTEF@5@5@+=feaafiart1ev1aaatCvAUfKttLearuWrP9MDH5MBPbIqV92AaeXatLxBI9gBaebbnrfifHhDYfgasaacH8akY=wiFfYdH8Gipec8Eeeu0xXdbba9frFj0=OqFfea0dXdd9vqai=hGuQ8kuc9pgc9s8qqaq=dirpe0xb9q8qiLsFr0=vr0=vr0dc8meaabaqaciaacaGaaeqabaqabeGadaaakeaacuWGnbqtgaqcaaaa@2DDF@ found by RIT always includes the genes found by UDE testing. Hence, RIT always has at least as much statistical power as a UDE test.

Typically, one needs to use two different independence tests with RIT, since the class variable *Y *is different from the genes *X*_*i*_. For simplicity, in our simulations (below) we have used the well-known Student's t-test for *X*_*i *_⊥ *Y *| ∅ and Fisher's z-transformation for testing *X*_*i *_⊥ *X*_*j *_| ∅. The t-test is optimal (unbiased most powerful) for gaussian marginal distributions [15], but on the other hand is correct only for these distributions, which constitute a subset of larger PCWT distribution class (see Additional file 1). Fisher's z is consistent regardless of distribution. While the gaussian assumption is frequently made in gene expression analysis, for example in gene network inference [16], we emphasize that RIT is itself not restricted to this class. As an example, we applied RIT to 100 samples drawn from the non-linear distribution in figure 1B using the distribution-free Kolmogorov-Smirnov test [17] for *X*_*i *_⊥ *Y *and the Spearman Rank Test [17] for *X*_*i *_⊥ *X*_*j*_, both at the 5% level. This produced the correct result (*S*_1 _= {*X*_2_}, *S*_2 _= {*X*_1_}) in 99 runs out of 100 (the single error made was a false positive *X*_2 _in *S*_1_). Applying same tests to the distribution in figure 1C gave no significant findings, since this distribution is not in the PCWT class and therefore not detectable by RIT.

### Consistency of the RIT algorithm

Remarkably, the comparatively simple RIT algorithm can be shown to be *consistent *for any PCWT distribution; that is, as sample size increases, the RIT output M^
 MathType@MTEF@5@5@+=feaafiart1ev1aaatCvAUfKttLearuWrP9MDH5MBPbIqV92AaeXatLxBI9gBaebbnrfifHhDYfgasaacH8akY=wiFfYdH8Gipec8Eeeu0xXdbba9frFj0=OqFfea0dXdd9vqai=hGuQ8kuc9pgc9s8qqaq=dirpe0xb9q8qiLsFr0=vr0=vr0dc8meaabaqaciaacaGaaeqabaqabeGadaaakeaacuWGnbqtgaqcaaaa@2DDF@ converges to the set of MDE genes *M*. To prove this, note that RIT constructs a path from *Y *to each gene *X*_*k *_in the graph whose edges (*i*, *j*) correspond to the pairwise marginal dependencies *X*_*i *_⊥
 MathType@MTEF@5@5@+=feaafiart1ev1aaatCvAUfKttLearuWrP9MDH5MBPbIqV92AaeXatLxBI9gBaebbnrfifHhDYfgasaacH8akY=wiFfYdH8Gipec8Eeeu0xXdbba9frFj0=OqFfea0dXdd9vqai=hGuQ8kuc9pgc9s8qqaq=dirpe0xb9q8qiLsFr0=vr0=vr0dc8meaabaqaciaacaGaaeqabaqabeGadaaakeaacuGHLkIxgaGcaaaa@2E6E@*X*_*j *_| ∅ (i.e., the graph in figure 2B). The following theorem states that for PCWT distributions, the set of genes M^
 MathType@MTEF@5@5@+=feaafiart1ev1aaatCvAUfKttLearuWrP9MDH5MBPbIqV92AaeXatLxBI9gBaebbnrfifHhDYfgasaacH8akY=wiFfYdH8Gipec8Eeeu0xXdbba9frFj0=OqFfea0dXdd9vqai=hGuQ8kuc9pgc9s8qqaq=dirpe0xb9q8qiLsFr0=vr0=vr0dc8meaabaqaciaacaGaaeqabaqabeGadaaakeaacuWGnbqtgaqcaaaa@2DDF@ reachable through such paths coincides with the set *M *defined in equation (2).

**Theorem 1 ***For any PCWT data distribution, the set of MDE genes M is identical to the set of genes *M^
 MathType@MTEF@5@5@+=feaafiart1ev1aaatCvAUfKttLearuWrP9MDH5MBPbIqV92AaeXatLxBI9gBaebbnrfifHhDYfgasaacH8akY=wiFfYdH8Gipec8Eeeu0xXdbba9frFj0=OqFfea0dXdd9vqai=hGuQ8kuc9pgc9s8qqaq=dirpe0xb9q8qiLsFr0=vr0=vr0dc8meaabaqaciaacaGaaeqabaqabeGadaaakeaacuWGnbqtgaqcaaaa@2DDF@ = {*X*_*k *_∈ *X*} *for which there exists a sequence *Z1m
 MathType@MTEF@5@5@+=feaafiart1ev1aaatCvAUfKttLearuWrP9MDH5MBPbIqV92AaeXatLxBI9gBaebbnrfifHhDYfgasaacH8akY=wiFfYdH8Gipec8Eeeu0xXdbba9frFj0=OqFfea0dXdd9vqai=hGuQ8kuc9pgc9s8qqaq=dirpe0xb9q8qiLsFr0=vr0=vr0dc8meaabaqaciaacaGaaeqabaqabeGadaaakeaacqWGAbGwdaqhaaWcbaGaeGymaedabaGaemyBa0gaaaaa@3069@ = {*Z*_1_,..., *Z*_*m*_} ⊆ *X between Z*_1 _= *Y and Z*_*m *_= *X*_*k *_*such that Z*_*i *_⊥
 MathType@MTEF@5@5@+=feaafiart1ev1aaatCvAUfKttLearuWrP9MDH5MBPbIqV92AaeXatLxBI9gBaebbnrfifHhDYfgasaacH8akY=wiFfYdH8Gipec8Eeeu0xXdbba9frFj0=OqFfea0dXdd9vqai=hGuQ8kuc9pgc9s8qqaq=dirpe0xb9q8qiLsFr0=vr0=vr0dc8meaabaqaciaacaGaaeqabaqabeGadaaakeaacuGHLkIxgaGcaaaa@2E6E@*Z*_*i*+1 _| ∅, *i *= 1,..., *m *- 1.

The full proof of this theorem is given in Additional file 1. If the data distribution is not PCWT, the theorem may not hold; figure 1C shows a typical counter-example. Assuming that the independence tests used are consistent, consistency of the RIT algorithm immediately follows from the above. This result is a good argument in favor of RIT, since consistency is a widely accepted *necessary *condition for a sound statistical procedure [15]. To our knowledge, no other algorithm for detecting MDE genes has been proven to be consistent.

### Biological relevance of the PCWT class

Next, we will show that the PCWT class is a reasonable model for gene expression data (or, more generally, for any measurements of biological systems). Since cellular systems are believed to be well described by complex networks [18], it is reasonable to assume that the distribution of *all *variables *X' *comprising a cellular network (transcripts, proteins, metabolites, etc.) can be modelled as a *Bayesian network *[19]. The following theorem, given by [20], asserts that the PCWT class contains all data distributions associated with such networks.

**Theorem 2 ***Any strictly positive distribution faithful to a Bayesian network is PCWT*.

However, we typically cannot measure all variables *X'*, but merely a subset *X*; for example, with microarrays we can perhaps measure most transcripts but no proteins or metabolites. Unfortunately, this means that in many cases *X *cannot be modelled by a Bayesian network [21]. Nevertheless, the next theorem asserts that *X *is still PCWT.

**Theorem 3 ***Let X' be a random vector with a PCWT distribution and let S, T be any two disjoint subsets of the components of X'. Then with probability 1, the distribution of X *= (*X' *\ {*S*, *T*} | *T *= *t*) *is also PCWT*.

The proof is found in theorems 5 and 6 of [22]. Theorem 3 states that for PCWT distributions, we may fix some variables *T *to constant values *t *and ignore other variables *S*, and the remaining variables will still form a PCWT distribution. Thus, given that the distribution of all variables *X' *comprising the cellular network are PCWT, then any measurements *X *we make will also have a PCWT distribution, even though we fail to measure many variables of the system and perhaps fix others to constant values by experimental design. We therefore conclude that PCWT is a realistic distribution class for biological data.

### Multiplicity and FDR control

Consistency is an asymptotic result however, and is still far from satisfactory for the small sample sizes typical for microarray data. Due to the large amounts of tests made, it is necessary to properly adjust for multiplicity, or else many findings are likely to be false positives. This issue has been thoroughly investigated for univariate tests [1], but our situation is more complicated since RIT performs multiple iterations of testing, and also chooses which tests to make in each iteration depending on the outcome of the previous one.

To ensure multiplicity control, we employ an induction argument. Fix an *α *∈ [0, 1]. Assume as the induction hypothesis that in the first **foreach **loop of the algorithm (figure 2A) we have tested the null hypotheses H0i
 MathType@MTEF@5@5@+=feaafiart1ev1aaatCvAUfKttLearuWrP9MDH5MBPbIqV92AaeXatLxBI9gBaebbnrfifHhDYfgasaacH8akY=wiFfYdH8Gipec8Eeeu0xXdbba9frFj0=OqFfea0dXdd9vqai=hGuQ8kuc9pgc9s8qqaq=dirpe0xb9q8qiLsFr0=vr0=vr0dc8meaabaqaciaacaGaaeqabaqabeGadaaakeaacqWGibasdaqhaaWcbaGaeGimaadabaGaemyAaKgaaaaa@303B@ = *X*_*i *_⊥ *Y *| ∅ for each *X*_*i *_and obtained p-values *p*_*i *_for each *X*_*i*_. We then sort these to obtain the order statistics *p*_(1) _≤ *p*_(2) _≤ ... *p*_(*n*)_, and apply a correction procedure to choose a gene set *S *(a "top list") with corrected p-values p˜
 MathType@MTEF@5@5@+=feaafiart1ev1aaatCvAUfKttLearuWrP9MDH5MBPbIqV92AaeXatLxBI9gBaebbnrfifHhDYfgasaacH8akY=wiFfYdH8Gipec8Eeeu0xXdbba9frFj0=OqFfea0dXdd9vqai=hGuQ8kuc9pgc9s8qqaq=dirpe0xb9q8qiLsFr0=vr0=vr0dc8meaabaqaciaacaGaaeqabaqabeGadaaakeaacuWGWbaCgaacaaaa@2E24@_*i *_satisfying

*P*(p˜
 MathType@MTEF@5@5@+=feaafiart1ev1aaatCvAUfKttLearuWrP9MDH5MBPbIqV92AaeXatLxBI9gBaebbnrfifHhDYfgasaacH8akY=wiFfYdH8Gipec8Eeeu0xXdbba9frFj0=OqFfea0dXdd9vqai=hGuQ8kuc9pgc9s8qqaq=dirpe0xb9q8qiLsFr0=vr0=vr0dc8meaabaqaciaacaGaaeqabaqabeGadaaakeaacuWGWbaCgaacaaaa@2E24@_*i *_≤ *α *| H0i
 MathType@MTEF@5@5@+=feaafiart1ev1aaatCvAUfKttLearuWrP9MDH5MBPbIqV92AaeXatLxBI9gBaebbnrfifHhDYfgasaacH8akY=wiFfYdH8Gipec8Eeeu0xXdbba9frFj0=OqFfea0dXdd9vqai=hGuQ8kuc9pgc9s8qqaq=dirpe0xb9q8qiLsFr0=vr0=vr0dc8meaabaqaciaacaGaaeqabaqabeGadaaakeaacqWGibasdaqhaaWcbaGaeGimaadabaGaemyAaKgaaaaa@303B@) ≤ *α*, *X*_*i *_∈ *S*.

This requirement is slightly weaker than FWER control, and is satisfied by the FDR-controlling procedure of Benjamini and Hochberg [23] (see Additional file 1 for a proof), which we employ in this paper. Other FDR-controlling procedures could probably also be used for obtaining *S*, but we have not attempted to prove (3) in the general case.

Now consider the recursive calls RIT(*X *\ *S*, *X*_*i*_). For each *X*_*i *_∈ *S*, this will test the null hypotheses H0ij
 MathType@MTEF@5@5@+=feaafiart1ev1aaatCvAUfKttLearuWrP9MDH5MBPbIqV92AaeXatLxBI9gBaebbnrfifHhDYfgasaacH8akY=wiFfYdH8Gipec8Eeeu0xXdbba9frFj0=OqFfea0dXdd9vqai=hGuQ8kuc9pgc9s8qqaq=dirpe0xb9q8qiLsFr0=vr0=vr0dc8meaabaqaciaacaGaaeqabaqabeGadaaakeaacqWGibasdaqhaaWcbaGaeGimaadabaGaemyAaKMaemOAaOgaaaaa@3198@ = *X*_*i *_⊥ *X*_*j *_| ∅ for every *X*_*j *_∉ *S*, producing the p-values *p*_*ij*_. We now combine the previously obtained p˜
 MathType@MTEF@5@5@+=feaafiart1ev1aaatCvAUfKttLearuWrP9MDH5MBPbIqV92AaeXatLxBI9gBaebbnrfifHhDYfgasaacH8akY=wiFfYdH8Gipec8Eeeu0xXdbba9frFj0=OqFfea0dXdd9vqai=hGuQ8kuc9pgc9s8qqaq=dirpe0xb9q8qiLsFr0=vr0=vr0dc8meaabaqaciaacaGaaeqabaqabeGadaaakeaacuWGWbaCgaacaaaa@2E24@_*i *_with these *p*_*ij *_to obtain a single p-value *p*_*j *_for each *X*_*j *_∉ *S*. To accomplish this, note that by theorem 1 *X*_*j *_∉ *M *is possible at this point only if, for *every X*_*i *_∈ *S*, either H0i
 MathType@MTEF@5@5@+=feaafiart1ev1aaatCvAUfKttLearuWrP9MDH5MBPbIqV92AaeXatLxBI9gBaebbnrfifHhDYfgasaacH8akY=wiFfYdH8Gipec8Eeeu0xXdbba9frFj0=OqFfea0dXdd9vqai=hGuQ8kuc9pgc9s8qqaq=dirpe0xb9q8qiLsFr0=vr0=vr0dc8meaabaqaciaacaGaaeqabaqabeGadaaakeaacqWGibasdaqhaaWcbaGaeGimaadabaGaemyAaKgaaaaa@303B@*or *H0ij
 MathType@MTEF@5@5@+=feaafiart1ev1aaatCvAUfKttLearuWrP9MDH5MBPbIqV92AaeXatLxBI9gBaebbnrfifHhDYfgasaacH8akY=wiFfYdH8Gipec8Eeeu0xXdbba9frFj0=OqFfea0dXdd9vqai=hGuQ8kuc9pgc9s8qqaq=dirpe0xb9q8qiLsFr0=vr0=vr0dc8meaabaqaciaacaGaaeqabaqabeGadaaakeaacqWGibasdaqhaaWcbaGaeGimaadabaGaemyAaKMaemOAaOgaaaaa@3198@ holds true. Hence, the null hypothesis for *X*_*j *_is

H0j=∩i∈S(H0i∪H0ij).
 MathType@MTEF@5@5@+=feaafiart1ev1aaatCvAUfKttLearuWrP9MDH5MBPbIqV92AaeXatLxBI9gBaebbnrfifHhDYfgasaacH8akY=wiFfYdH8Gipec8Eeeu0xXdbba9frFj0=OqFfea0dXdd9vqai=hGuQ8kuc9pgc9s8qqaq=dirpe0xb9q8qiLsFr0=vr0=vr0dc8meaabaqaciaacaGaaeqabaqabeGadaaakeaacqWGibasdaqhaaWcbaGaeGimaadabaGaemOAaOgaaOGaeyypa0ZaaqbuaeaacqGGOaakcqWGibasdaqhaaWcbaGaeGimaadabaGaemyAaKgaaOGaeSOkIuLaemisaG0aa0baaSqaaiabicdaWaqaaiabdMgaPjabdQgaQbaakiabcMcaPaWcbaGaemyAaKMaeyicI4Saem4uamfabeqdcqWIPissaOGaeiOla4caaa@4364@

This situation is known in statistics as *intersection-union *testing [24,25]. By the intersection-union method, a level *α *test for H0j
 MathType@MTEF@5@5@+=feaafiart1ev1aaatCvAUfKttLearuWrP9MDH5MBPbIqV92AaeXatLxBI9gBaebbnrfifHhDYfgasaacH8akY=wiFfYdH8Gipec8Eeeu0xXdbba9frFj0=OqFfea0dXdd9vqai=hGuQ8kuc9pgc9s8qqaq=dirpe0xb9q8qiLsFr0=vr0=vr0dc8meaabaqaciaacaGaaeqabaqabeGadaaakeaacqWGibasdaqhaaWcbaGaeGimaadabaGaemOAaOgaaaaa@303D@ is

reject H0j
 MathType@MTEF@5@5@+=feaafiart1ev1aaatCvAUfKttLearuWrP9MDH5MBPbIqV92AaeXatLxBI9gBaebbnrfifHhDYfgasaacH8akY=wiFfYdH8Gipec8Eeeu0xXdbba9frFj0=OqFfea0dXdd9vqai=hGuQ8kuc9pgc9s8qqaq=dirpe0xb9q8qiLsFr0=vr0=vr0dc8meaabaqaciaacaGaaeqabaqabeGadaaakeaacqWGibasdaqhaaWcbaGaeGimaadabaGaemOAaOgaaaaa@303D@ if ∃_*i *_∈ *S *: max {*p*_*i*_, *p*_*ij*_} ≤ *α*/|*S*|,

and the corresponding p-value *p*_*j *_is computed as

pj=|S|min⁡i(max⁡{pi,pij}).
 MathType@MTEF@5@5@+=feaafiart1ev1aaatCvAUfKttLearuWrP9MDH5MBPbIqV92AaeXatLxBI9gBaebbnrfifHhDYfgasaacH8akY=wiFfYdH8Gipec8Eeeu0xXdbba9frFj0=OqFfea0dXdd9vqai=hGuQ8kuc9pgc9s8qqaq=dirpe0xb9q8qiLsFr0=vr0=vr0dc8meaabaqaciaacaGaaeqabaqabeGadaaakeaacqWGWbaCdaWgaaWcbaGaemOAaOgabeaakiabg2da9iabcYha8jabdofatjabcYha8naaxababaGagiyBa0MaeiyAaKMaeiOBa4galeaacqWGPbqAaeqaaOGaeiikaGIagiyBa0MaeiyyaeMaeiiEaGNaei4EaSNaemiCaa3aaSbaaSqaaiabdMgaPbqabaGccqGGSaalcqWGWbaCdaWgaaWcbaGaemyAaKMaemOAaOgabeaakiabc2ha9jabcMcaPiabc6caUaaa@4C8A@

The factor |*S*| derives from a Bonferroni correction for the outer intersection in (4). This completes the induction step; as the induction hypothesis is easily satisfied in the first round of testing, it follows by induction that with these corrections, RIT always yields p-values. Finally, the Benjamini-Hochberg procedure may be applied again to control the false discovery rate. Alternatively, more stringent measures such as family-wise error rate control [26] may be used, if desired. Formal proofs of the correctness of each of the above steps can be found in Additional file 1. A detailed pseudocode of RIT implementing each step is given in Additional file 3.

### Simulated data

To illustrate the above result and also to assess the statistical power of RIT as a function of the sample size, we conducted a simulation study. To this end, we designed a distribution with multivariate differential expression, chosen so that 10% of the genes were MDE, but only half of these (5%) were UDE and thus detectable a univariate test (see methods section for details). We compared the performance of RIT against a typical univariate test, namely Student's t-test [15] with FDR correction [23], and also against the popular Recursive Feature Elimination (RFE) feature selection method [14].

**Figure 3:**
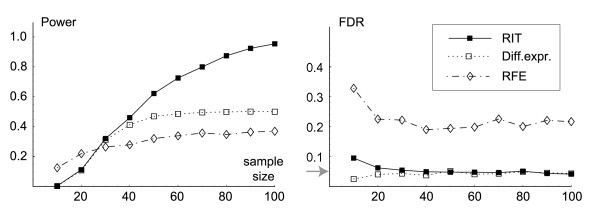
**Simulation results**. Simulation results for the RIT algorithm, differential expression using the t-test, and RFE. Left, statistical power (1 – false negative rate) as a function of sample size. Right, false discovery rate (FDR) as a function of sample size. Grey arrow marks the nominal FDR = 0.05.

Figure 3 summarizes the results of this experiment. We find that RIT does indeed control the FDR at the nominal level (*α *= 0.05), in the same way as the univariate test. The power of the univarate test converges to 0.5 as expected (since only half of the MDE genes were UDE), while the RIT converges to 1.0, in agreement with our theoretical results. Thus, when multivariate effects are present, RIT affords more power than the univariate test at the same FDR level. In contrast, the RFE method clearly does not control the FDR, choosing many genes unrelated to *Y*. RFE also displays low power, most likely because it considers some MDE genes to be "redundant" for prediction and consequently ignores these. Similar behavior is to be expected from other feature selection methods, as explained above. A second simulation study using a different distribution was also performed, with similar results (see Additional file 2). We conclude that it is feasible to apply the RIT algorithm to small-sample data while controlling the FDR at the desired level. Exact sample size requirements cannot be inferred from figure 3 however, as this depends on the data distribution, in particular the fraction of MDE genes and the amount of noise.

### Microarray data

We next tested the RIT algorithm on two publicly available microarray data sets (see methods section). The diabetes data contrasts pancreas islets expression from normal *vs. *type 2 diabetic patients [27]. The original study identified 370 genes as UDE, but this did not account for multiplicity. The q-value method declared only the top three genes of the original study to be significant: *Arnt*, *Cdc14a*, and *Ddx3y*. The RIT algorithm identified an additional 21 transcripts as MDE, of which 5 were unknown EST:s and 16 were known genes according to the NCBI Gene database [28]. We would like to stress that RIT is an hypotheses-generating method, and that the discovered MDE genes may or may not be of *functionally *related to the target variable. Nevertheless, we conducted a literature validation of the 16 known genes (table 1) to search for possible biologically important findings. Five of these (31%) were previously associated with diabetes. Among the remaining 11 novel genes, several give rise to interesting hypotheses: for example, *Dopey1 *was recently shown to be active in the vesicle traffic system, the mechanism that delivers insulin receptors to the cell surface. Four genes encoded transcription factors, as do the majority of previously discovered diabetes-associated genes [27]. The *Usp9y *gene discovered by RIT is associated with male infertility and Sertoli cell-only syndrome. Interestingly, so is the UDE *Ddx3Y *gene. This is unlikely to be a coincidence as only 6 human genes were annotated with this function in NCBI Gene. This is an example of general tendency we have observed in our experiments, that additional MDE genes discovered by RIT often are functionally related to the UDE genes, which is hardly surprising given that RIT relies on pairwise independence test. The chloride channels *Clca2 *and *Clcn1 *are also highly interesting findings, as ion channels in pancreas islets has been shown to regulate insulin secretion [29]. The diabetes-associated potassium ion channel *Kcng1 *was also discovered by RIT, strengthening this hypothesis.

**Table 1:** Diabetes-related genes. Genes with multivariate expression patterns discovered by the RIT algorithm for the diabetes data.

Gene	Status	Function	Ref. (PMID)
Bcat1	D	Candidate gene for the type I diabetes susceptibility locus Idd6.	14563018
Clca2	N	Chloride channel. Multiple functions, colocalizes with adhesion molecule integrin *β*_4_.	15707651
Clcn1	N	Chloride channel. Regulates electric excitability of the skeletal muscle membrane.	7735894
Cltb	D	Involved in insulin receptor internalization.	7821727
Dopey1	N	Involved in Golgi/ER vesicle traffic.	1630131
Epha7	N	Ephrin receptor subfamily. Ephrin receptors are associated with the pancreatic islets.	15681844
Hcap-G	N	Chromosome condensation protein, member of the condensin complex.	14593730
Kcng1	D	Potassium channel. Potassium channels are involved in regulation of insulin secretion.	16567526
Loc51152	N	Melanoma antigen.	
Psmal	D	Glutamate carboxypeptidase. Inhibition prevents long-term type 1-diabetic neuropathy.	11809162
Sox5P	N, T	Transcription factor activity (GeneOntology). Related to the sex determining region Y-gene.	
Sptlc2	N	Sphingolipid biosynthesis enzyme, upregulated upon transepidermal water loss.	12445191
Ssx2	N, T	Suggested role as transcriptional repressor.	12007189
Tfap2B	D, T	Genetic variations in this gene are associated with type 2 diabetes.	15940393
Usp9Y	N	Associated with male infertility and Sertoli cell-only (SCO) syndrome.	12871878
Zf	N, T	Specific inhibitor of the transcription factor Luman. Inhibits herpes virus replication.	16282471

Status column: D, diabetes-related; N, novel; T, transcription factor.

The breast cancer data contrasts good-prognosis *vs. *poor-prognosis patients [30]. This set had large amounts of pairwise correlations among genes, resulting in a highly connected dependence graph. To limit the number of findings in this case, we required significant correlations to exceed a threshold 0.85 to be considered by RIT (see discussion). The original study identified a set of 70 cancer-related genes. In addition to these, the RIT algorithm identified 43 MDE genes. Literature validation revealed that 23 of these (53%) had a previously known function in cancer development, whereof 6 (14%) were specifically implicated in breast cancer (table 2). An additional 10 transcripts (23%) were cell cycle-related and may also be considered as potential cancer proliferation genes. Our literature validation thus confirmed 39 (77%) of the genes reported by RIT to be cancer-related. The higher percentage in this case as compared to the diabetes data may reflect the fact that more genes are known for cancer than for diabetes. To assessed the amount of cancer annotations among the 5,000 genes considered, we examined an additional 43 genes chosen at random. Indeed, we found that about 25% of all genes had some cancer or cell cycle-related annotation. Nevertheless, the above fraction of 77% validated genes is highly significant with a Binomial tail p-value < 10^-20^.

**Table 2:** Breast cancer-related genes. Genes with multivariate expression patterns discovered by the RIT algorithm for the breast cancer data.

Gene	Status	Function	Ref. (PMID)
Anln	C	Over-expressed in diverse common human tumors, may have potential as biomarker.	16203764
Aurka	B	Cell cycle-regulated kinase, possible prognostic indicator for patients with breast tumors.	12833450
Aurkb	C	Highly expressed in high-grade gliomas, correlated with malignancy and clinical outcomes.	15072448
Birc5	B	Prevents apoptotic cell death, differentially expressed in breast cancer.	16142334
Blm	C	Cooperates with p53 in regulation of cell growth, associated with colorectal cancer.	11781842, 12242432
Brrn1	CC	Required for the conversion of interphase chromatin into condensed chromosomes.	
Bub1	C	Mutations in this gene have been associated with aneuploidy and several forms of cancer.	15931389
Ccnb1	B	Detected in various human breast cancer cell lines and breast tumor tissues.	11779217
Ccnb2	CC	Essential component of the cell cycle regulatory machinery.	
Cdc20	C	Regulatory protein in the cell cycle, associated with gastric cancer.	15701830
Cdc25A	C	Known oncogene, required for progression from G1 to the S phase of the cell cycle.	14673957
Cdc45L	CC	Important for early steps of DNA replication in eukaryotes, loss may affect cell proliferation.	9660782
Cdca8	CC	Required for stability of the bipolar mitotic spindle.	
Depdc1	N	Contains domain of unknown function often present together with the RhoGAP domain.	
Depdc1B	N	Unknown function, has conserved RhoGAP domain (GTPase-activator protein).	
Dlg7	C	Potential oncogenic target of AURKA, may play a role in human carcinogenesis.	15987997, 12527899
Exo1	C	Possible cancer predisposing gene.	15328369
Fam64A	N	Unknown function.	
Fbxo5	CC	Function in ubiquitination, inhibits the anaphase promoting complex.	
Foxm1	C	Stimulates the proliferation of tumor cells.	16489016
Gpr116	N	Has two immunoglobulin-like repeats, may have a role in the immune system.	12435584
H2Afz	C	Chromatin remodeling at the c-myc oncogene involves the local exchange of this histone.	15878876
Hist1H1B	N	Histone protein.	
Hist1H1E	N	Histone protein.	
Hist1H4B	N	Histone protein.	
Kif20A	C	Required for cytokinesis, related to AURKB. Likely to be involved in pancreatic cancer.	15263015, 15665285
Kif23	CC	Interacts with CYK4, to form the centralspindlin complex. Essential for cell division.	11782313
Kif2C	CC	Important for anaphase chromosome segregation.	
Kifc1	CC	Involved in localization of PLK1, AURKB, and CDC14A during anaphase.	15263015
Mad2L1	C	Mitotic checkpoint gene, involved mainly in colorectal carcinogenesis.	12970887
Nek2	B	Significantly up-regulated in breast carcinomas.	15492258
Pitrm1	N	Novel member of the metalloendoprotease superfamily.	10360838
Prr11	N	Unknown function. Predicted interactions with E2F, which is involved in cancer.	16437386
Pttg2	C	Potent oncogene, expressed at high levels in various human tumors and tumor cell lines.	10806349
Racgap1	B	Implicated in in breast cancer cell proliferation	15863513
Rad54L	B	Candidate oncosupressor in breast or colon carcinomas, lymphomas and meningiomas.	12614485
Spbc25	CC	Essential kinetochore component, significant role in mitotic events	14699129
Stil	C	Involved in mitosis and in increased mitotic activity in tumor cells.	
Tk1	C	Marker for non-small cell lung cancer. May be important in epithelial ovarian cancer.	15809747, 11992400
Tpx2	C	May be important in both progression lung cancer, possible prognostic predictor.	16489064
Ttk	CC	Required for centrosome duplication and for the normal progression of mitosis.	15618221, 14657364
Ube2C	C	Required for destruction of mitotic cyclins. Highly expressed in human primary tumors.	12874022
Ube2S	N	Essential component of the ubiquitin-protein conjugation system.	15454246

Status column: B, Breast cancer-specific; C, Cancer-related; CC, Cell cycle-related; N, novel.

Encouraged by the above result, we set out to investigate the remaining 10 genes that were not previously associated with cancer. We found three histone proteins, which may be related to chromatin remodelling. One novel gene *Gpr116 *was recently identified as a G-protein with a potential role in immune response. The novel gene *Prr11 *had predicted binding sites for the transcription factor E2F, which in turn is known to be crucial in the control of tumorigenesis. *Ube2s *is an essential component of the ubiquitin-protein conjugation system, which is implicated in various cancer forms. This gene is also functionally related to the known cancer gene *Ube2c*, which also was discovered by RIT. Also interesting were the novel proteins *Depdc1 *and *Depdc1b*, both containing RhoGAP domains. This may implicate them in the regulation of various Rho GTPases, which are currently being investigated as cancer-therapy targets [31].

## Discussion

At a first glance, RIT might seem similar to existing algorithms for "local" network inference around "seed genes" [32,33]. However, network inference is a much harder problem than detecting MDE, and typically requires testing for *conditional *independence (while RIT requires only marginal independence tests). Consequently, these algorithms require substantially larger samples and stronger distribution assumptions, and their time-complexity is exponential with respect to the number of genes found [33].

RIT is less useful for data with large and strongly correlated transcriptional changes, such as the breast cancer data set considered herein. For cancer data, even the (smaller) fraction of UDE genes has been estimated to be on the order of 50% of all genes [34], and the set of MDE genes is presumably much larger. Thus, the concept of MDE is simply not very useful in this case, since most genes turn out to be MDE. Thus, a principled approach for prioritizing among all these genes is urgently needed. For the cancer data, we let RIT prioritize the findings by considering stronger correlations to be more important. This seems reasonable, and we were able to confirm the end results in this case against the literature. However, this problem is ultimately unsolvable by statistical methods, and must instead be addressed by integrating other kinds of information. A possible step towards a principled solution building upon the present work would be to combine the independence tests used here with other data sources and prior beliefs (perhaps in the form of Bayesian probabilities) to guide the RIT algorithm towards more "interesting" genes.

It is important to realize that RIT does not perform feature selection in the usual machine learning-sense: feature selection aims to find the set of features (genes) optimal for constructing an accurate predictor of the target variable, while RIT aims to find the MDE genes, which need not be optimal for prediction.

These are two different problems, and they should be treated separately.

In this study we have limited ourselves to two-class data. However, it is straightforward to extend the RIT algorithm to find multivariate expression patterns with other types of target variables, such as multiple classes data or continuous target variables such as survival times. To accomplish this, only the independence tests used need to be replaced. This "modularity" is a useful property of RIT: to handle different situations, it is sufficient to "plug in" different independence tests. For example, a continuous target variable could be handled by using the Fisher z-transformation also for testing *X*_*j *_⊥ *Y*. More complex, non-linear independence relations may be handled using nonparametric tests such as the Kolmogorov-Smirnov test [17] or kernel-based tests [35]. However, a basic limitation of the RIT algorithm is that at least one gene must be UDE for any MDE genes to be found. This is an inherent property of the PCWT class.

Dynamic (time-series) data could also be considered, although some additional assumptions may be necessary to ensure PCWT distributions in this case. For example, assuming a Markov condition, time-series data can be modelled using Dynamic Bayesian Networks (DBNs) [19]. The DBN methodology essentially transforms a dynamic model over *n *nodes into an ordinary BN over 2*n *nodes. Thus, DBNs also result in PCWT distributions as described herein (albeit of twice the dimensionality) and RIT is therefore applicable to detecting multivariate changes in dynamic as well as in static data.

## Conclusion

The RIT algorithm is a principled, general approach that increases the power of small-sample, genome-wide expression studies by considering not only univariate differential expression but also multivariate effects. In contrast to previous approaches which focus on testing gene sets [5-7,10,11], RIT gives a p-value for each gene and provides control over false positive findings in terms of individual genes. RIT may be very useful in situations where little univariate differential expression is observed, as exemplified by the diabetes data experiment.

## Methods

### Simulation study

In our simulations, we used a multivariate gaussian distribution with *n *= 1, 000 genes and |*M*| = 100 MDE genes, of which |*U*| = 50 were differentially expressed. We first designed a 4-dimensional gaussian distribution with a class-dependent mean vector *μ*_*y *_= 2*y*·(0, 0, 1, 1) and covariance matrix

Σ=4⋅(2111122212551258),
 MathType@MTEF@5@5@+=feaafiart1ev1aaatCvAUfKttLearuWrP9MDH5MBPbIqV92AaeXatLxBI9gBaebbnrfifHhDYfgasaacH8akY=wiFfYdH8Gipec8Eeeu0xXdbba9frFj0=OqFfea0dXdd9vqai=hGuQ8kuc9pgc9s8qqaq=dirpe0xb9q8qiLsFr0=vr0=vr0dc8meaabaqaciaacaGaaeqabaqabeGadaaakeaacqqHJoWucqGH9aqpcqaI0aancqGHflY1daqadaqaauaabeqaeqaaaaaabaGaeGOmaidabaGaeGymaedabaGaeGymaedabaGaeGymaedabaGaeGymaedabaGaeGOmaidabaGaeGOmaidabaGaeGOmaidabaGaeGymaedabaGaeGOmaidabaGaeGynaudabaGaeGynaudabaGaeGymaedabaGaeGOmaidabaGaeGynaudabaGaeGioaGdaaaGaayjkaiaawMcaaiabcYcaSaaa@4431@

equal for both classes. We then constructed the full distribution for the 100 MDE genes using 25 of these 4-blocks. The remaining features had the same covariance matrix but had mean *μ *= (0, 0, 0, 0). We varied the sample size as 10, 20, 30,..., 100.

The Recursive Feature Elimination (RFE) procedure was implemented as described [14], eliminating 20% of the genes in each iteration. We used the radius-margin bound proposed by [36] as a goodness measure for choosing the optimal gene set.

### Microarray data sets

The diabetes data set is from the study by Gunton et al. [27] and is publicly available at the Diabetes Genome Anatomy Project [37]. This data set contrasts human pancreas islets expression from normal (n = 7) *vs. *type 2 diabetic (n = 5) patients. The original data comprises 44,928 probesets from the Affymetrix U133A and B chips. We used only the A chip in our experiments, since we needed to evaluate our results against literature and the A chip contains better annotated sequences. Moreover, since initial analysis using the full A chip resulted in no significant findings, we reduced multiplicity by pre-filtering genes by variance, keeping only the 5,000 most variable genes.

The breast cancer data set consist of 78 samples from patients divided into one "good prognosis" group (*n *= 44) and one a "poor prognosis" group (*n *= 34) based on the time until relapse [30]. The data set is freely available from Rosetta Inpharmatics [38]. The arrays used contains approx. 25,000 transcripts, out of which 4,918 were selected using the same quality filter as in the original publication.

## Authors' contributions

RN devised the multiple comparison procedure, performed the simulation study and the application to the microarray data sets, performed most of the literature validation, and wrote most of the manuscript. JMP devised the basic idea for the RIT algorithm and the wrote the proof of theorem 1. JB assisted with the literature validation. JT participated in study design and co-wrote the manuscript.

## Supplementary Material

Additional File 1 **Supplementary information**. This supplement contains details on the PCWT class, proofs for theorems and an additional simulation study. Click here for file

Additional File 2 **Supplementary figure 1**. Describes the second simulation study. Click here for file

Additional File 3 **Supplementary figure 2**. Contains the pseudocode for the RIT algorithm with p-value corrections. **Software availability**. The RIT algorithm is available as a Mathematica notebook upon request. Click here for file
