# Supplementary material for: Detecting multivariate differentially expressed genes
Source: BMC Bioinformatics. 2007 May 9;8:150. doi: 10.1186/1471-2105-8-150 (PMC1885271; doi:10.1186/1471-2105-8-150)
Supplement: Additional File 1 — Supplementary information. This supplement contains details on the PCWT class, proofs for theorems and an additional simulation study. [file 1471-2105-8-150-S1.pdf]

Detecting multivariate differential expression patterns

Supplementary information

This document provides supplementary information for the paper "Detecting multivariate expression patterns" by Nilsson, Peña, Björkegren and Tegnér.

1 The PCWT class

The PCWT class of distributions considered in the paper is defined by three probability axioms. Below, we consider a random vector X as a set of variables $\{X_1, \ldots X_n\}$, and let capital letters R, S, T, U denote any disjoint subsets of those variables, while $X_k \in X$ denotes a single variable. The notation $S \perp T | R$ denotes the conditional independence $P(S, T \mid R) = P(S \mid R)P(T \mid R)$.

Definition 1 *The PCWT class is defined as the set of distributions that satisfy the following probability axioms:*

Strict Positivity: $P(S) > 0$

Composition: $S \perp T | R \wedge S \perp U | R \implies S \perp T \cup U | R$

Weak Transitivity: $S \perp T | R \wedge S \perp T | R \cup X_k \implies S \perp X_k | R \vee X_k \perp T | R$

A thorough treatment of these axioms can be found in e.g. Pearl (1988). Its relevance for biological data, in particular microarray data, is discussed in the main paper.

2 Proof of theorem 1

We here give the full proof of theorem 1 of the main paper, which asserts that if the independence tests used are correct (which holds for any consistent test in the sample limit), the RIT algorithm is correct. First we remind the reader of the definition of multivariate differential expression (MDE) as given in equation (2) of the main paper:

Definition 2 *The set of MDE genes are those X_i satisfying*

$$M = \{X_i : \exists S \subseteq X : X_i \not\perp Y \mid S\}.$$

For the proof of theorem 1, we will need the following well-known properties, given by Pearl (1988).

Theorem 1 *Let R, S, T, U denote any disjoint subsets of the variables X. Any probability distribution satisfies the following properties:*

$$\begin{aligned}
&\textit{Symmetry:} \quad S \perp T | R \implies T \perp S | R \\
&\textit{Decomposition:} \quad S \perp T \cup U | R \implies S \perp T | R \\
&\textit{Weak union:} \quad S \perp T \cup U | R \implies S \perp T | R \cup U \\
&\textit{Contraction:} \quad S \perp T | R \cup U \wedge S \perp U | R \implies S \perp T \cup U | R
\end{aligned}$$

Theorem 2 (Main paper theorem 1) *For any PCWT data distribution, the set of MDE genes M is identical to the set of genes $\hat{M} = \{X_k \in X\}$ for which there exists a sequence $Z_1^m = \{Z_1, \ldots, Z_m\} \subseteq X$ between $Z_1 = Y$ and $Z_m = X_k$ such that $Z_i \not\perp Z_{i+1} | \emptyset$, $i = 1, \ldots, m-1$.*

Proof: Let $\hat{M}^c = X \setminus \hat{M}$ and fix any $X_k \in \hat{M}^c$. Since $Y \perp X_k | \emptyset$ and $X_i \perp X_k | \emptyset$ for any $X_i \in \hat{M}$, we have $\{Y\} \cup \hat{M} \perp \hat{M}^c | \emptyset$ by the composition property. Then $Y \perp X_k | S$ for any $S \subset X \setminus \{X_k, Y\}$ by the weak union and decomposition properties, so $X_k \notin M$.

For the converse, fix any $X_k \in \hat{M}$ and let $Z_1^m = \{Z_1, \ldots, Z_m\}$ be a shortest sequence between $Z_1 = Y$ and $Z_m = X_k$ such that $Z_i \not\perp Z_{i+1} | \emptyset$ for $i = 1, \ldots, m-1$. Then we must have $Z_i \perp Z_j | \emptyset$ for $j > i+1$, or else a shorter sequence would exist. We will prove that $Z_1 \not\perp Z_m | Z_2^{m-1}$ for *any* such shortest sequence, by induction over the sequence length m. The case $m = 2$ is trivial. Consider the case $m = p$. By the induction hypothesis, for any $i < p$ and any chain Z_1^i we have $Z_1 \not\perp Z_i | Z_2^{i-1}$. By construction of the sequence,

$$Z_1 \perp Z_i | \emptyset, \quad 3 \leq i \leq p \quad \underset{\text{(composition)}}{\implies} \quad Z_1 \perp Z_3^i | \emptyset \tag{1}$$

$$\underset{\text{(weak union)}}{\implies} \quad Z_1 \perp Z_i | Z_3^{i-1}. \tag{2}$$

Now assume to the contrary that $Z_1 \perp Z_p | Z_2^{p-1}$. Together with (2), weak transitivity implies

$$Z_1 \perp Z_2 | Z_3^{p-1} \quad \vee \quad Z_2 \perp Z_p | Z_3^{p-1}.$$

The latter alternative contradicts the induction hypothesis. The former together with (1) implies $Z_1 \perp Z_2^{p-1} | \emptyset$ by contraction, which implies $Z_1 \perp Z_2 | \emptyset$ by decomposition. This is a contradiction; therefore we conclude that $Y \not\perp X_k | Z_2^{m-1}$, and thus $X_k \in M$. $\square$

3 FDR control

A p-value for a null hypothesis H_0 is a statistic p satisfying

$$P(p \leq \alpha \mid H_0) \leq \alpha.$$

In the RIT algorithm, we need to ensure that the p-values of the "top genes", selected according to some threshold, still satisfy this property. This is non-trivial due to multiplicity, which causes the "top" statistics $p_{(1)}, p_{(2)} \dots$ to be skewed towards zero even under H_0. The following lemma shows that the FDR-controlling procedure by Benjamini and Hochberg (1995) yields p-values.

Lemma 3 *Assume $p_1, \dots p_{n_0}$ are independent p-values corresponding to true null hypotheses $H_0^1, \dots H_0^{n_0}$, while $p_{n_0+1}, \dots p_n$ are p-values corresponding to the remaining false null hypotheses, taking any joint distribution on $[0,1]^{n-n0}$. Then the Benjamini-Hochberg procedure*

$$p'_{(i)} = \frac{np_{(i)}}{i}$$

satisfies

$$P(p'_{(i)} \leq \alpha \mid H_0^i) \leq \alpha$$

Proof: Since the $p_1, \dots p_{n_0}$ are independent, the $p_{(i)}|H_0^i$ are order statistics of a $U(0,1)$ distribution. These are well known to be beta distributed,

$$p_{(i)} \sim Beta(i, n-i+1),$$

and therefore

$$\begin{aligned} P(p'_{(i)} \leq \alpha \mid H_0) &= P(p_{(i)} \leq i\alpha/n \mid H_0) \\ &= I_{i\alpha/n}(i, n-i+1) \end{aligned}$$

where I_z is the regularized incomplete beta function. For all α, this function takes its largest value for $i = 1$, so it suffices to note that for all n,

$$I_{i\alpha/n}(1, n) = 1 - \left(1 - \frac{\alpha}{n}\right)^n \leq \alpha. \qquad \square$$

Note that the procedure by Storey (2003) with $\pi_0 = 1$ is identical to Benjamini-Hochberg, and so this can also be used. We believe that this lemma holds for other FDR-controlling procedures as well, although we have not attempted to find a general proof.

Having secured the p-value property, the following theorem formally establishes the induction step in the RIT algorithm. This basically shows that, when given a set of p-values in a previous iteration of RIT, the correction procedure used results in p-values also in the next iteration. Thus, by induction, the RIT algorithm always produces p-values. Below, we assume that the set S is selected using the above correction procedure so that we indeed have "proper" p-values throughout.

Theorem 4 *Assume that the distribution of* (X, Y) *is PCWT. For a given set* $S \subseteq X$ *and* $X_i \in S$*, let* p_i *be a p-value for the null hypothesis* $H_0^i : X_i \notin M$*. Choose an* $X_j \notin S$*, and let* p_{ij} *be p-values for* $H_0^{ij} = X_i \perp X_j \,|\, \emptyset$ *for each* $X_i \in S$*. Then the null hypothesis*

$$H_0^j = \bigcap_{i \in S} (H_0^i \cup H_0^{ij}) \tag{3}$$

holds true if $X_j \notin M$*, and*

$$p_j = |S| \min_{i \in S} (\max\{p_i, p_{ij}\})$$

is a p-value for H_0^j*.*

Proof: Since the data distribution is PCWT, we know from theorem 1 that

$$\exists X_i : X_i \in M \wedge X_i \not\perp X_j \mid \emptyset \implies X_j \in M$$

Negating this, we obtain

$$X_j \notin M \implies \forall X_i : X_i \notin M \vee X_i \perp X_j \mid \emptyset$$

Thus, equation (3) is a null hypothesis for $X_j \notin M$. Further, since p_i and p_{ij} are p-values, it holds that

$$P(p_i \leq \alpha \mid H_0^i) \leq \alpha \quad \text{and} \quad P(p_{ij} \leq \alpha \mid H_0^{ij}) \leq \alpha.$$

It is now easy to verify that

$$\begin{aligned} P(p_j \leq \alpha \mid H_0^j) &= P\left(|S| \min_{i \in S} (\max\{p_i, p_{ij}\}) \leq \alpha \mid \bigcap_{i \in S} (H_0^i \cup H_0^{ij}) \right) \\ &\leq \sum_{i \in S} P\left(\max\{p_i, p_{ij}\} \leq \alpha/|S| \mid H_0^i \cup H_0^{ij} \right) \\ &\leq \sum_{i \in S} P\left(p_i \leq \alpha/|S| \mid H_0^i \wedge p_{ij} \leq \alpha/|S| \mid H_0^{ij} \right) \\ &\leq \sum_{i \in S} \min\left\{ P(p_i \leq \alpha/|S| \mid H_0^i),\ P(p_{ij} \leq \alpha/|S| \mid H_0^{ij}) \right\} \\ &\leq |S| \cdot \alpha/|S| = \alpha \end{aligned}$$

which proves that p_j is a p-value for the null hypothesis (3). $\square$

We note that this theorem is similar to the results concerning intersection-union testing derived by Berger (1982). A summary of the RIT algorithm with these corrections for error rate control is given in supplementary figure 2.

4 Gaussian distributions and PCWT

We here provide a proof that the family of multivariate Gaussian distributions is contained in PCWT. This proof is essentially the same as that given by Studený (2004, pp. 36-37).

Theorem 5 *Any multivariate Gaussian $f(x) = N(x|\mu, \Sigma)$ distribution satisfies strict positivity, composition and weak transitivity.*

Proof: Strict positivity is immediate. As above, let R, S, T, U denote any disjoint subsets of the variables X. Let $\Sigma_{S\times T}$ denote the sub-matrix of Σ with rows corresponding to the variables in S and columns corresponding to T, and let $\Sigma_{S|T}$ be the (quadratic) covariance matrix of the conditional variable $S|T$. Then the composition property

$$S \perp T|R \wedge S \perp U|R \implies S \perp T \cup U|R$$

is equivalent to

$$(\Sigma_{ST|R})_{S\times T} = 0 \wedge (\Sigma_{SU|R})_{S\times U} = 0 \implies (\Sigma_{STU|R})_{S\times TU} = 0$$

for the Gaussian case. Noting that $(\Sigma_{STU|R})_{ST\times ST} = \Sigma_{ST|R}$ and similarly $(\Sigma_{STU|R})_{SU\times SU} = \Sigma_{SU|R}$ for any Gaussian (marginalizing out a variable does not change the remaining covariances) proves the above implication and thus proves the composition property. Similarly, the weak transitivity property

$$S \perp T|R \wedge S \perp T|R \cup X_k \implies S \perp X_k|R \vee X_k \perp T|R$$

is equivalent to

$$(\Sigma_{ST|R})_{S\times T} = 0 \wedge (\Sigma_{ST|RX_k})_{S\times T} = 0 \implies (\Sigma_{SX_k|R})_{S\times X_k} = 0 \vee (\Sigma_{X_kT|R})_{X_k\times T} = 0$$

Since every matrix in the above is conditioned on R, we may write $\Sigma' = \Sigma_{X\setminus R|R}$ and simplify this to

$$(\Sigma'_{ST})_{S\times T} = 0 \wedge (\Sigma'_{ST|X_k})_{S\times T} = 0 \implies (\Sigma'_{SX_k})_{S\times X_k} = 0 \vee (\Sigma'_{X_kT})_{X_k\times T} = 0$$

Because of the previously established composition property, it holds in general that

$$S \perp T|R \iff \forall X_i \in S, X_j \in T : X_i \perp X_j|R,$$

so that it suffices to prove the weak transitivity relation for all such pairs X_i, X_j, that is,

$$\sigma'_{ij} = 0 \wedge \sigma'_{ij|k} = 0 \implies \sigma'_{ik} = 0 \vee \sigma'_{kj} = 0.$$

Rewriting

$$\sigma'_{ij|k} = \sigma'_{ij} - \sigma'_{ik}\sigma'_{kj}/\sigma'_{kk} = 0 \iff \sigma'_{ij}\sigma'_{kk} = \sigma'_{ik}\sigma'_{kj}$$

and using $\sigma'_{ij} = 0$ gives the result. $\square$

5 Simulation with "large blocks"

The simulation provided in the main paper considers a situation with genes interacting in "blocks" of size 4. To investigate the performance of RIT with larger such blocks, we considered the case where both the MDE genes M and the remaining "irrelevant" genes $X \setminus M$ have banded covariance matrices,

$$\Sigma = \begin{pmatrix} 2 & -1 & & \\ -1 & 2 & \ddots & \\ & \ddots & \ddots & -1 \\ & & -1 & 2 \end{pmatrix}.$$

This results in two large "blocks". The mean vector μ were the same as in the first simulation. The result from this simulation is qualitatively similar (supplementary figure 1). RIT and the t-test still controls FDR as expected, while only RIT achieves 100% power asymptotically. RFE performs better here in terms of FDR, but still provides low power.

References

Yoav Benjamini and Yosef Hochberg. Controlling the false discovery rate: a practical and powerful approach to multiple testing. *J R Statist Soc B*, 57(1): 289–300, 1995.

Roger L. Berger. Multiparameter hypothesis testing and acceptance sampling. *Technometrics*, 24(4):295–300, November 1982.

Judea Pearl. *Probabilistic reasoning in intelligent systems.* Morgan Kauffman Publishers, Inc., San Fransisco, California, 1988.

John D. Storey. The positive false discovery rate: a bayesian interpretation and the q-value. *Annals of Statistics*, 31:2013–2035, 2003.

Milan Studený. *Probabilistic Conditional Independence Structures.* Springer, 1st edition, 2004.
