# Supplementary figures and images for: Detecting multivariate differentially expressed genes
Source: BMC Bioinformatics. 2007 May 9;8:150. doi: 10.1186/1471-2105-8-150 (PMC1885271; doi:10.1186/1471-2105-8-150)

Supplementary figure 1

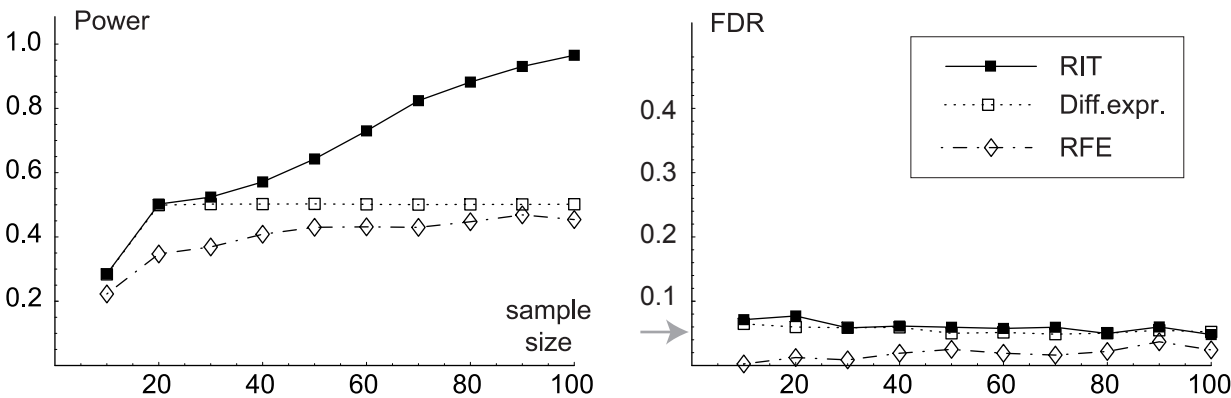

Supplement: Additional File 2 — Supplementary figure 1. Describes the second simulation study. [file 1471-2105-8-150-S2.pdf]
