# Supplementary material for: Detecting multivariate differentially expressed genes
Source: BMC Bioinformatics. 2007 May 9;8:150. doi: 10.1186/1471-2105-8-150 (PMC1885271; doi:10.1186/1471-2105-8-150)
Supplement: Additional File 3 — Supplementary figure 2. Contains the pseudocode for the RIT algorithm with p-value corrections. Software availability. The RIT algorithm is available as a Mathematica notebook upon request. [file 1471-2105-8-150-S3.pdf]

Supplementary figure 2

```
Function RIT(X, S, p, φ, α)
Input: data X, set S ⊂ X, p-values p, test φ, level α
foreach X_j ∉ S do
    foreach X_i ∈ S do
        Let p_ij = φ(X_i, X_j);
    end
    Let p_j = |S| min_i max{p_i, p_ij} ;                 // Suppl. Theorem 4
end
Let p_j = |X \ S| p_j / r_j ;                            // Suppl. Lemma 3
Let S' = {X_j ∉ S : p_j ≤ α};
if S' ≠ ∅ then
    p = RIT(X \ S, S', p, φ, α);
end
return p ;                                   // Result is modified vector p
```

Recursive Independence Test with FDR control. Assumes ϕ returns p-values. Start with $\mathrm{RIT}(X \cup \{Y\}, \{Y\}, 0, \phi, \alpha)$. Here p is a vector of p-values corresponding to the element of X.
